# Relationship between Traditional Chinese Medicine Syndrome Elements and Prognosis of Patients with IgA Nephropathy

**DOI:** 10.1155/2022/2270406

**Published:** 2022-07-30

**Authors:** Jianghua Ke, Shuwei Duan, Linchang Liu, Zhiwei Yin, Shuang Li, Yujing Ke, Jin Yao, Ying Zheng, Weiguang Zhang, Yilun Qu, Ping Li, Zheyi Dong, Xiangmei Chen

**Affiliations:** ^1^National Clinical Research Center for Kidney Diseases, State Key Laboratory of Kidney Diseases, Beijing Key Laboratory of Kidney Disease Research, First Medical Center of Chinese PLA General Hospital, Nephrology Institute of the Chinese People's Liberation Army, Beijing 100853, China; ^2^School of Clinical Medicine, Guangdong Pharmaceutical University, Guangzhou 510006, China

## Abstract

**Objective:**

It is not clear which Traditional Chinese Medicine- (TCM-) related elements affect primary IgA nephropathy (IgAN) progression. Here, we explored the risk factors, based on TCM syndrome elements, related to the prognosis of primary IgAN patients.

**Methods:**

We analyzed patients with newly diagnosed, biopsy-proven IgAN at a single institution from December 2013 to September 2021. Basic clinical and pathological characteristics were assessed at the time of renal biopsy. The study endpoint was end-stage renal disease (ESRD: eGFR <15 ml/min per 1.73 m^2^, dialysis, or kidney transplantation) and/or eGFR decreased by >30% from baseline. Kaplan‒Meier survival analysis was used to explore the role of TCM syndrome elements in IgAN progression. Multivariate Cox regression analysis with adjustment for traditional risk factors was performed to explore TCM syndrome elements that may influence patient prognosis. The factors correlated with TCM syndrome elements in IgAN patients were further evaluated by logistic regression analysis.

**Results:**

During a median follow-up of 22.0 months, 53 (12.5%) of the 423 included IgAN patients reached the study endpoint. The main IgAN disease location elements were the kidney, liver, and spleen. The main IgAN disease nature elements were Yin-deficiency and Qi-deficiency, dampness, Yang-deficiency, phlegm, and Blood-deficiency. Kaplan‒Meier analysis identified three disease locations (liver, spleen, and kidney) and four disease natures (Qi-deficiency, Yang-deficiency, phlegm, and dampness) as elements associated with poor renal survival in IgAN patients. In multivariate Cox regression analysis, baseline Yang-deficiency was an independent risk predictor of poor prognosis in primary IgAN patients (hazard ratio 2.338; 95% confidence interval [CI]: 1.208–4.525; *P*=0.012) after adjustment for traditional risk factors. Furthermore, logistic regression analysis identified being female (odds ratio [OR] 2.518; 95% CI: 1.538–4.122; *P* < 0.001), older age (OR 1.043; 95% CI: 1.022–1.065; *P* < 0.001), low hemoglobin levels (OR 0.984; 95% CI: 0.971–0.996; *P*=0.013), and cellular/fibrocellular crescents (OR 1.706; 95% CI: 1.068–2.728; *P*=0.026) as factors affecting Yang-deficiency in IgAN patients.

**Conclusions:**

Yang-deficiency independently predicts the risk of poor prognosis in primary IgAN patients. Being female, older age, low hemoglobin levels, and cellular/fibrocellular crescents were independently associated with Yang-deficiency in IgAN patients.

## 1. Introduction

IgA nephropathy (IgAN) is the most common primary glomerular disease in the world [1, 2], with an incidence of more than 2.5 cases per 100,000 people [3]. It is also the main cause of chronic kidney disease and end-stage renal disease (ESRD) [1, 4], placing a heavy economic and social burden on countries and individuals. The clinical and pathological manifestations of IgAN patients are diverse, and individual prognosis varies greatly. Early detection of risk factors for disease progression is of great significance for delaying the development of disease and improving the prognosis of patients.

At present, the Kidney Disease International Global Outcomes (KDIGO) guidelines [4] recommend that the international prediction tool [5] be used to assess the progression risk of IgAN patients. The internal verification accuracy of the prediction model reaches 80%, and the external verification [6, 7] also shows good prediction ability. However, in about 25% of patients, it will develop into renal insufficiency within 10 years even in those with clinically early IgAN. Consequently, there is still a lack of more risk factors for IgAN.

Traditional Chinese Medicine (TCM) uses syndrome differentiation and treatment as the core and has unique advantages in delaying the progress of IgAN [8–10]. Syndrome differentiation used the basic theory of TCM to elucidate the nature of the disease through its external expression. It is also the premise of treatment (legislation, prescription, and medication), which is highly empirical and subjective, and is not conducive to quantitative diagnosis.

TCM syndrome element differentiation is a new syndrome differentiation system put forward by professors Wenfeng Zhu and Yongyan Wang [11–13], which facilitates quantification of TCM syndrome differentiation [14–16]. TCM syndrome elements are the indecomposable diagnostic units that make up the syndrome, with independent connotation, including disease location syndrome elements, such as the heart, liver, or spleen, and disease nature syndrome elements, such as Qi deficiency, blood stasis, or phlegm.

In the past, numerous studies have investigated the prognosis of IgAN in the context of TCM, mostly focusing on exploring the prognostic differences between different syndrome types [17, 18]. However, it was unclear which TCM-related risk factors affected the progress of IgAN. In this study, we explored the impact of TCM syndrome elements on the prognosis of IgAN.

## 2. Materials and Methods

### 2.1. Participants

**Figure 1:**
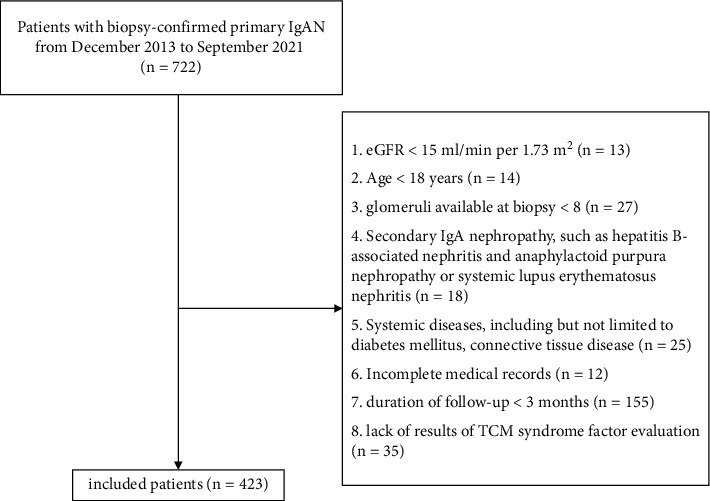
Flow diagram of the study.

Overall, 722 patients with primary IgA nephropathy, newly diagnosed by renal biopsy in the first medical center of the Chinese People's Liberation Army General Hospital from December 2013 to September 2021, were retrospectively screened. The inclusion criteria were as follows: (1) estimated glomerular filtration rate (eGFR) ≥15 ml/min per 1.73 m^2^ at the time of renal biopsy; (2) age 18 years or older at the time of renal biopsy, regardless of sex; and (3) availability of records of the four diagnoses of TCM. The exclusion criteria were as follows: (1) fewer than eight glomeruli available at biopsy; (2) secondary IgA nephropathy, such as hepatitis B-associated nephritis, anaphylactoid purpura nephropathy, systemic lupus erythematosus nephritis, and so on; (3) systemic diseases, such as diabetes, connective tissue disease, and so on; (4) patients with follow-up of less than 90 days; (5) lack of results of TCM syndrome factor evaluation; and (6) incomplete medical records. According to the inclusion and exclusion criteria, 423 patients were included in the analysis (Figure 1). This study was in accordance with the Declaration of Helsinki and approved by the Research Ethics Committee of the first medical center of the Chinese People's Liberation Army General Hospital (Approval no. S2021-165-01), and all subjects had signed an informed consent.

### 2.2. Clinical Data

Baseline clinical and laboratory data were collected at the time of renal biopsy. This information included sex, age, body mass index (BMI), mean arterial pressure (MAP) that was calculated as diastolic blood pressure (BP) + 1/3 (systolic BP − diastolic BP), total serum protein, serum albumin (ALB), blood urea nitrogen(BUN), serum creatinine, serum uric acid (SUA), eGFR, calculated by the Chronic Kidney Disease Epidemiology Collaboration equation adjusted for Asian populations [19], total cholesterol, triglycerides, high-density lipoprotein cholesterol (HDL-C), low-density lipoprotein cholesterol (LDL-C), 24 h urinary protein, hemoglobin (Hb), urine red blood cell count (URBC), and the use of renin-angiotensin system blocker (RASB) and immunosuppression. The use of RASB, including angiotensin-converting enzyme inhibitors and angiotensin-receptor blockers, and immunosuppression were defined as any exposure to these treatments at or prior to kidney biopsy.

### 2.3. Evaluation of Renal Histopathological Damage

The pathological diagnosis was confirmed by two pathologists and was reevaluated according to the Oxford classification [20]. Both pathologists were blinded to the clinical data. When the two pathologists' scores differed, a third pathologist rescored the images. A final score was decided after discussion among three pathologists. The Oxford classification: mesangial hypercellularity, a mesangial score ≤0.5 was recorded as M0, a mesangial score >0.5 was recorded as M1; endocapillary hypercellularity, E0: absent or E1: present; segmental glomerulosclerosis or adhesion, S0: absent or S1: present; tubular atrophy/interstitial fibrosis, T0 ≤ 25%, T1: 26%–50%, or T2 > 50%; and cellular/fibrocellular crescents, C0: absent, C1 < 25% or C2 ≥ 25% of glomeruli.

### 2.4. Primary Outcome Definitions

The primary outcome was a composite event including the first occurrence of either a 30% decline in eGFR from the value at biopsy or end-stage renal disease (ESRD: eGFR <15 ml/min per 1.73 m^2^, dialysis, or kidney transplantation).

### 2.5. Traditional Chinese Medicine Materials

We collected the TCM four diagnosis within 7 days before and after renal biopsy, including symptoms, signs, tongue, and pulse. TCM syndrome elements included disease location syndrome elements, such as the heart, liver, or spleen, and disease nature syndrome elements, such as Qi deficiency, blood stasis, or phlegm. TCM syndrome element was diagnosed according to the “Syndrome element differentiation” compiled by Professor Wenfeng Zhu, which involved identifying the syndrome element and the score of the syndrome element corresponding to each symptom in the syndrome differentiation factor scale. Each symptom was divided into no, mild, moderate, and severe grade (4), the main complaint or severe symptom, the score multiplied by 1.5; or moderate symptom, the score multiplied by 1; or mild symptom, the score multiplied by 0.7. When the sum of the score of the TCM for diagnosis of the syndrome element reaches or exceeds 100 points, these syndrome elements can be diagnosed.

### 2.6. Statistical Analysis

Statistical analysis was performed using SPSS version 26.0 (IBM SPSS Corp., Armonk, NY, USA). Continuous variables were expressed as the mean ± standard deviation for normally distributed data, as the median of the interquartile range (IQR) (25%, 75%) for nonnormally distributed data, and the constituent ratio of qualitative data was expressed as frequency (percentage). Comparisons between groups were performed using Student's *t*-test, Mann–Whitney *U* test, or chi-squared test. The cumulative renal survival rate was calculated using Kaplan‒Meier survival curves, which were compared by log-rank test. Multivariate Cox proportional hazard regression models were used to analyze the factors affecting the prognosis of patients with IgAN. Multivariate logistic regression was used to analyze the correlation between TCM syndrome factors and clinicopathological indexes. A *p*-value <0.05 was considered statistically significant (two-sided).

## 3. Results

### 3.1. Clinical and Pathological Characteristics of Patients with IgA Nephropathy

Of the 722 patients screened, 423 patients with biopsy-proven IgAN met the selection criteria and were included in this study (Figure 1). The clinical and pathological characteristics of the patients are summarized in Table 1. According to the KDIGO 2021 clinical practice guideline [4], patients were classified into five chronic kidney disease (CKD) stages by eGFR levels.

There were 155 (21.5%) patients with <90 days' follow-up who were excluded. Baseline comparisons of the 423 patients including those followed up for <90 days were shown in Supplementary Table S1 and Table S2. There were no significant differences in TCM syndrome elements or clinical and pathological characteristics between the included and excluded groups.

After a median follow-up of 22.0 months (IQR 6.0, 56.0), 53 (12.5%) patients experienced the primary outcome, including 31 (7.3%) patients with a 30% decrease in eGFR and 22 (5.2%) patients who developed ESRD.

### 3.2. Distribution of Syndrome Elements of Patients with IgA Nephropathy

A total of 423 patients with IgAN were assessed for eight syndrome elements of disease location. Kidney was the most common location, followed by liver and spleen, while the lung, heart, stomach, Biao, and Jifu were less often involved (Table 2). Eleven syndrome elements of the disease nature of 423 patients were listed in Table 2. Yin-deficiency and Qi-deficiency had the highest frequency, followed by dampness, Yang-deficiency, phlegm, Blood-deficiency, with Blood-stasis, heat, water retention, Qi-depression, and Yang-hyperactivity being less frequent.

### 3.3. Kaplan–Meier Curves of Cumulative Renal Survival by Traditional Chinese Medicine Syndrome Elements

**Figure 2:**
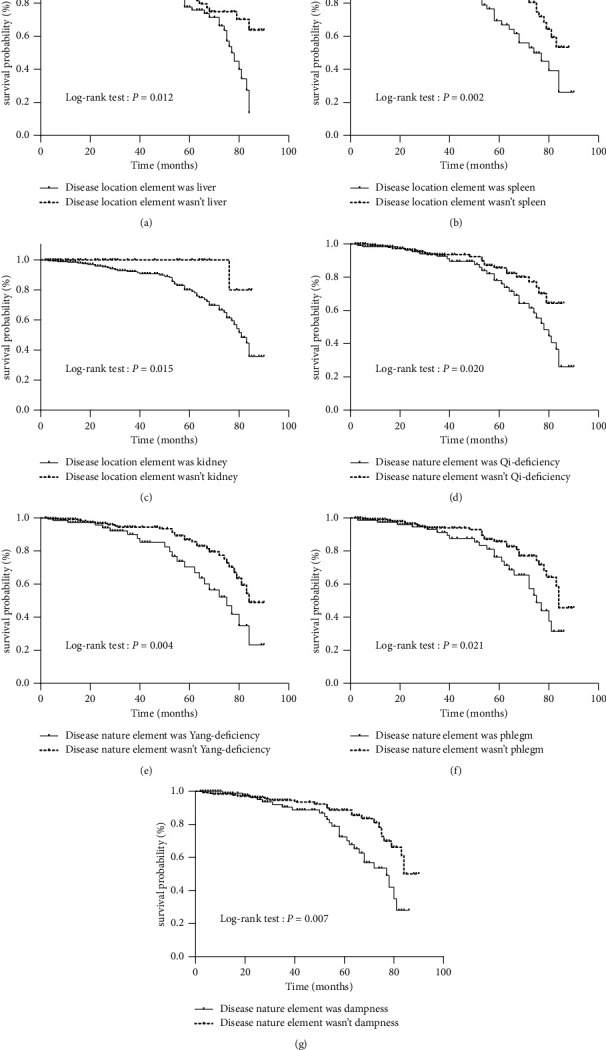
Kaplan–Meier analysis for the primary outcome of TCM syndrome elements. (a) Kidney survival rates in the disease location element was liver and nonliver group. (b) Kidney survival rates in the disease location element was spleen and nonspleen group. (c) Kidney survival rates in the disease location element was kidney and non-kidney group. (d) Kidney survival rates in the disease nature element was Qi-deficiency and non-Qi-deficiency group. (e) Kidney survival rates in the disease nature element was Yang-deficiency and non-Yang deficiency group. (f) Kidney survival rates in the disease nature element was phlegm and nonphlegm group. (g) Kidney survival rates in the disease nature element was dampness and nondampness group.

Kaplan‒Meier analysis indicated that there were seven syndrome elements that were related to poor renal survival in IgAN patients, including three disease location elements and four disease nature elements. The disease location elements were liver, spleen, and kidney, while the disease nature elements were Qi-deficiency, Yang-deficiency, phlegm, and dampness. The difference was statistically significant at *P* < 0.05 (Figure 2).

### 3.4. Risk Factors for Kidney Prognosis in IgAN Patients

Multivariate Cox proportional hazard regression analysis was performed to explore the risk factors that may influence the prognosis of patients with IgAN. Traditional risk factors (age, MAP, eGFR, proteinuria, immunosuppressant use, RASB use, and MEST scores) as core variables were included using the entry, according to the International IgA Nephropathy Prediction Tool, recommended by KDIGO guidelines [5]. TCM syndrome elements with *P* < 0.1 during Kaplan‒Meier analysis, including heart, liver, spleen, and kidney of syndrome elements of disease location and Qi-deficiency, blood-deficiency, Yang-deficiency, dampness, phlegm, water retention, and Yang-hyperactivity of syndrome elements of the disease nature, were analyzed using forward selection (LR). Multivariate Cox proportional hazard regression analysis indicated that baseline Yang-deficiency, eGFR, 24 h urinary protein, and T2 were independent risk factors for IgAN progression (Table 3).

### 3.5. Analysis of Factors Influencing Yang-Deficiency in Patients with IgA Nephropathy

Since Yang-deficiency was an independent risk factor affecting the prognosis of IgAN patients, we further performed univariate and multivariate logistic regression analyses to explore the factors correlated with Yang-deficiency in patients with IgAN. Univariate logistic regression analysis showed that being female, older age, endocapillary hypercellularity, cellular/fibrocellular crescents, higher levels of HDL-C and URBC, and a lower level of ALB, SUA, and Hb were statistically significantly associated with Yang-deficiency (*P* < 0.05, Table 4). It suggested that patients with Yang-deficiency had a more severe pathological lesion than those without Yang-deficiency. After the variables were screened, the major factors identified included being female, older age, lower Hb levels, and cellular/fibrocellular crescents (*P* < 0.05, Table 4).

And we analyzed the relationship between the Yang-deficiency in patients with CKD stages or 24 h urinary protein at the time of renal biopsy, two important characteristics of patients with IgAN. The 423 patients were divided into Yang-deficiency group (147, 34.8%) and non-Yang-deficiency group (276, 65.2%). At the time of renal biopsy, there were no statistical differences between the two groups in eGFR, CKD stages and 24 h urinary protein (Table S3).

## 4. Discussion

In this study, using TCM syndrome element analysis of IgAN, the main disease location elements of IgAN were kidney, liver, and spleen, while the main disease nature elements of IgAN were Yin-deficiency and Qi-deficiency, dampness, Yang-deficiency, phlegm, and Blood-deficiency. Yang-deficiency was identified as an independent risk factor for poor prognosis of IgAN. The factors related to Yang deficiency were being female, older, having a low Hb level, and cellular/fibrocellular crescents.

With a view of additional risk factors, we adjusted the prognostic factors of IgAN, as recommended by international guidelines [4]; we found that Yang-deficiency was still an independent risk factor for poor prognosis in IgAN patients. Previous studies had also reported that a Yang-deficiency constitution is related to the prognosis in IgAN patients [17]. The urinary protein level was the highest, the renal function was the worst, and the pathological damage was the most severe in IgAN patients with spleen‒kidney Yang-deficiency syndrome [18, 21]. Huangqi Guizhi Wuwu decoction, a classic prescription, with the functions of supplementing Qi, warming Yang, removing blood stasis, and dredging collaterals, had also been proved to alleviate proteinuria and protect renal function in IgAN patients with spleen‒kidney Yang-deficiency syndrome [22]. Traditional Chinese medicine believed that Yang Qi can stimulate and promote the physiological functions of human tissues and organs and promote the production and operation of blood, as well as the production, distribution, and excretion of body fluid. Yang-deficiency is mainly located in the spleen, kidney, and heart, and kidney Yang-deficiency is generally considered to be the most important. Deficiency of kidney Yang weakens the promotion of the production and the movement of blood; then it leads to anemia. Similarly, deficiency of kidney Yang was unable to promote the production, distribution, and excretion of body fluid; then the urine volume decreases and edema is observed clinically.

This study found that the proportion of patients with Yang-deficiency syndrome was higher among female than among male patients with IgAN. Previous studies have also shown that women are more common in the population with Yang-deficiency [23, 24], and that being female is positively correlated with Yang-deficiency [25]. In TCM, men and women are considered to have different endowments of Yin and Yang. Men have more Yang and less Yin, while women have more Yin and less Yang. Women prefer more stillness and less movement, which can easily lead to Yang-deficiency. At the same time, Yang-deficiency in men is considered to be a manifestation of more serious disease, which is consistent with previous studies that found that the prognosis of male patients with IgAN was worse [26–28].

In this study, age was a positive predictor of Yang-deficiency in patients with IgAN, which is consistent with the TCM theory that human Yang decreases with increasing age. Chen et al. used a multicenter, epidemiological, on-the-spot investigation to study the TCM syndromes of 1,016 patients with IgAN [18]. They found that, with increased age, the proportion of patients with spleen‒lung Qi-deficiency syndrome decreased, while the proportion of spleen‒kidney Yang-deficiency syndrome increased [18]. Tang et al. proposed that, with the increase in age, the proportion of individuals with liver and kidney Yin (Qi)-deficiency syndrome decreased, while the proportion of those with spleen and kidney (Qi) Yang-deficiency syndrome increased [29]. In addition, IgAN usually occurs in young adults. With increasing age, the course of the disease was prolonged. Over a long period of time, true Yang can be damaged, which may also be one of the factors leading to the positive correlation between age and IgAN Yang-deficiency.

In this study, the Hb level of patients with Yang-deficiency was lower. Previous studies also found that the level of hemoglobin in Yang-deficiency syndrome group was significantly lower than that in Qi-deficiency syndrome group and Qi-Yin deficiency syndrome group [30]. Hb is the basis for the diagnosis of anemia. Renal anemia is not only a common complication in patients with middle and late stage of CKD, but is also evidence of a poor prognosis in patients with Yang-deficiency.

Anemia in TCM belongs to Blood-deficiency syndrome, Yin-deficiency syndrome, and so on. Yang-deficiency leads to insufficient gasification, which affects the movement of water valley essence in the spleen and stomach and then affects the process of blood production and transformation, resulting in Blood-deficiency. Qi is invisible, blood is the carrier of qi, and qi can be lost with the loss of blood, so blood deficiency can further aggravate qi deficiency. In TCM, Qi belongs to Yang; blood belongs to Yin. Yin and Yang are fundamental and can be transformed into each other, and Yin-deficiency can further lead to Yang-deficiency.

Exploring the correlation between pathological changes and TCM syndrome elements belongs to the microscopic syndrome differentiation. Because all pathological products are tangible, they are all positive, according to the theory of TCM. Thus, there is a theory of micro-syndrome differentiation without deficiency syndrome. Because of its attribute of growth, the formation of crescent can belong to Yang [31]. Previous studies have found that the deficiency syndrome of IgAN with crescent formation is mainly spleen‒kidney Yang-deficiency syndrome [32]. We also found that cellular/fibrocellular crescents were positively correlated with Yang-deficiency in patients with IgAN, but the correlation between Yang-deficiency and crescent formation needs to be confirmed in a larger patient population.

In this study, eGFR and T lesions remained significant independent risk factors for a poor prognosis of IgAN in multivariate Cox regression analysis. However, MAP, M, E, and S lesions and the use of immunosuppressants were not statistically significantly associated in multivariate analysis. This was consistent with the results of multivariate Cox regression analysis of the full model, including race, of the International IgA Nephropathy Prediction Tool derivation cohort [5].

However, our results in terms of other indicators differed from those of this full model with race included. In this study, 24-hour urinary protein was an independent risk factor for poor prognosis in IgAN patients, while RASB and age were not statistically significant in multivariate Cox regression analysis. The quantity of urinary protein has been shown to be associated with poor prognosis in many studies [33–37]. The timing for renal biopsy in the patients included in this study ranged from 2013 to 2021, and the use rate of RASB inhibitors was 89.1%, which was significantly higher than that (32.4%) of patients included in the International IgA Nephropathy Prediction Tool derivation cohort, which had a median timing of renal biopsy of 2006. The 2012 KDIGO guidelines [38] recommend that patients with IgAN be routinely treated with RASB during or after biopsy. Due to the widespread use of RASB, its effect on prognosis is underestimated. Some previous studies had not found that RASB has an independent prognostic effect on IgAN [39, 40]. The effect of age on the prognosis of IgAN patients remains controversial [28, 33, 41, 42]. In this study, no effect of age on the prognosis of IgAN was found, which is related to the inclusion of age-related Yang-deficiency in the model.

This study had some limitations. First, this study was a single-center retrospective study, with an insufficient number of included patients. Second, the median follow-up period of 22 months was relatively short.

## 5. Conclusion

This study found that Yang-deficiency syndrome should be an independent risk factor for poor prognosis of IgAN. In turn, Yang-deficiency syndrome was related to sex, age, hemoglobin, and cellular/fibrocellular crescents. The combination of TCM syndrome elements and clinicopathological indexes may improve the clinical curative effect and delay the progress of IgAN to a certain extent.

## Figures and Tables

**Table 1:** Clinical and pathological characteristics of patients with IgA nephropathy [median (IQR)/*n* (%)].

Characteristics	Value (*n* = 423)
Baseline	
Male (%)	243 (57.4)
Age (years)	37.0 (30.0–44.0)
BMI (kg/m^2^)	24.5 (22.0–27.1)
MAP (mmHg)	97.3 (89.3–106.7)
Total serum protein (g/L)	65.5 (60.6–70.3)
ALB (g/L)	39.3 (36.0–42.1)
Blood urea nitrogen (mmol/L)	5.7 (4.5–7.0)
SUA (umol/L)	372.5 (305.6–449.8)
Total cholesterol (mmol/L)	4.3 (3.7–5.0)
Triglycerides (mmol/L)	1.6 (1.1–2.2)
HDL-C (mmol/L)	1.1 (0.9–1.3)
LDL-C (mmol/L)	2.7 (2.3–3.3)
Hb (g/L)	134.0 (119.0–148.0)
URBC (HPF)	9.0 (3.5–22.5)
Serum creatinine (umol/L)	99.5 (77.0–126.0)
eGFR (ml/min per 1.73 m^2^)	80.6 (57.2–103.0)
CKD, *n* (%)	
1	160 (37.8)
2	145 (34.3)
3a	64 (15.1)
3b	37 (8.8)
4	17 (4.0)
24 h urinary protein (g/24 h)	1.2 (0.7–2.0)
<0.5, *n* (%)	54 (12.8)
0.5–1, n (%)	117 (27.7)
1–2, n (%)	141 (33.3)
2–3, n (%)	53 (12.5)
≥3, n (%)	58 (13.7)
Oxford classification (%)	
M1	185 (43.7)
E1	84 (19.9)
S1	310 (73.3)
T1	116 (27.4)
T2	81 (19.1)
C1	114 (27.0)
C2	5 (1.2)
Immunosuppressant use (%)	231 (54.6)
RASB use (%)	377 (89.1)

IQR: interquartile range; BMI, body mass index; MAP: mean arterial pressure; ALB: albumin; SUA: serum uric acid; HDL-C: high-density lipoprotein cholesterol; LDL-C: low-density lipoprotein cholesterol; Hb: hemoglobin; URBC: urine red blood cell count; eGFR: estimated glomerular filtration rate; CKD: chronic kidney disease; M: mesangial hypercellularity; E: endocapillary hypercellularity; S: segmental glomerulosclerosis or adhesion; T: tubular atrophy/interstitial fibrosis; C: cellular/fibrocellular crescents; and RASB: renin-angiotensin system blocker.

**Table 2:** Distribution of syndrome elements in IgAN patients (*n* (%)).

Syndrome elements of disease location	Frequency	Syndrome elements of disease nature	Frequency
Kidney	371 (87.7)	Yin-deficiency	334 (79.0)
Liver	212 (50.1)	Qi-deficiency	204 (48.2)
Spleen	154 (36.4)	Dampness	154 (36.4)
Lung	46 (10.9)	Yang-deficiency	147 (34.8)
Heart	30 (7.1)	Phlegm	144 (34.0)
Stomach	10 (2.4)	Blood-deficiency	136 (32.2)
Biao	8 (1.9)	Blood-stasis	65 (15.4)
Jifu	7 (1.7)	Heat	53 (12.5)
		Water retention	37 (8.7)
		Qi-depression	36 (8.5)
		Yang-hyperactivity	23 (5.4)

**Table 3:** Multivariate Cox proportional hazard regression analysis on the prognosis of IgAN patients.

Variables	Multivariate cox regression		
	HR	95% CI	*P*
Yang-deficiency	2.338	1.208–4.525	0.012
Age	1.006	0.967–1.046	0.781
MAP	1.009	0.984–1.035	0.465
M	1.200	0.625–2.302	0.584
E	0.670	0.279–1.607	0.369
S	0.975	0.430–2.213	0.952
T	—	—	0.090
T0	—	—	—
T1	1.546	0.556–4.304	0.404
T2	2.716	1.009–7.316	0.048
Immunosuppressant use	0.643	0.325–1.271	0.204
RASB use	1.137	0.397–3.257	0.812
eGFR	0.983	0.969–0.997	0.019
24 h urinary protein	1.199	1.022–1.407	0.026

MAP: mean arterial pressure; M: mesangial hypercellularity; E: endocapillary hypercellularity; S: segmental glomerulosclerosis or adhesion; T: tubular atrophy/interstitial fibrosis; RASB: renin-angiotensin system blocker; and eGFR: estimated glomerular filtration rate.

**Table 4:** Univariate and multivariate logistic regression analysis of factors associated with Yang-deficiency among IgAN patients.

Variables	Univariate logistic regression			Multivariate logistic regression		
	OR	95% CI	*P*	OR	95% CI	*P*
Female (vs. male)	3.120	2.060–4.725	＜0.001	2.419	1.483–3.946	＜0.001
Age	1.041	1.018–1.064	＜0.001	1.048	1.023–1.073	＜0.001
BMI	0.991	0.942–1.043	0.729			
MAP	0.991	0.976–1.006	0.257			
Total serum protein	0.977	0.953–1.002	0.070			
ALB	0.934	0.899–0.970	＜0.001			
Blood urea nitrogen	1.006	0.938–1.078	0.877			
Serum creatinine	0.999	0.995–1.003	0.533			
SUA	0.998	0.996–1.000	0.023			
Total cholesterol	1.080	0.912–1.280	0.373			
Triglycerides	0.873	0.734–1.037	0.123			
HDL-C	2.091	1.198–3.649	0.009			
LDL-C	1.175	0.933–1.479	0.170			
eGFR	0.997	0.991–1.004	0.418			
Urinary protein	1.070	0.949–1.207	0.269			
Hb	0.972	0.962–0.983	＜0.001	0.983	0.971–0.996	0.011
URBC	1.019	1.007–1.030	0.001			
Immunosuppressant use	1.170	0.782–1.751	0.445			
RASB use	1.400	0.712–2.751	0.329			
M1(vs. M0)	0.765	0.509–1.148	0.196			
E1(vs. E0)	1.743	1.072–2.835	0.025			
S1(vs. S0)	1.130	0.716–1.784	0.601			
T0	1.000	Reference	0.851			
T1	0.872	0.543–1.400	0.571			
T2	0.946	0.555–1.611	0.837			
C1/C2 (vs. C0)	1.980	1.281–3.061	0.002	1.845	1.154–2.949	0.010

BMI: body mass index; MAP: mean arterial pressure; ALB: albumin; SUA: serum uric acid; HDL-C: high-density lipoprotein cholesterol; LDL-C: low-density lipoprotein cholesterol; Hb: hemoglobin; URBC: urine red blood cell count; eGFR: estimated glomerular filtration rate; M: mesangial hypercellularity; E: endocapillary hypercellularity; S: segmental glomerulosclerosis or adhesion; T: tubular atrophy/interstitial fibrosis; C: cellular/fibrocellular crescents; and RASB: renin-angiotensin system blocker.

## Data Availability

The data presented in this study are available from the corresponding author upon request.

## Supplementary Materials

Supplementary Materials
*Table S1*. Baseline clinical and pathological characteristics of the 423 patients included and 155 patients followed <90 days. *Table S2*. Distribution of syndrome elements in the 423 patients included and 155 patients followed <90 days. *Table S3*. Distribution of CKD stages and 24 h urinary protein for patients at the time of renal biopsy with Yang-deficiency and non-Yang-deficiency.
