# Supplementary material for: Relationship between Traditional Chinese Medicine Syndrome Elements and Prognosis of Patients with IgA Nephropathy
Source: Evid Based Complement Alternat Med. 2022 Jul 30;2022:2270406. doi: 10.1155/2022/2270406 (PMC9356779; doi:10.1155/2022/2270406)
Supplement: Supplementary Materials — Table S1. Baseline clinical and pathological characteristics of the 423 patients included and 155 patients followed <90 days. Table S2. Distribution of syndrome elements in the 423 patients included and 155 patients followed <90 days. Table S3. Distribution of CKD stages and 24 h urinary protein for patients at the time of renal biopsy with Yang-deficiency and non-Yang-deficiency. [file 2270406.f1.docx]

TABLE S1: Baseline clinical and pathological characteristics of the 423 patients included and 155 patients followed＜90 days[median (IQR)/ n (%)].

| Characteristics | n=423 | n=155 | *P* |
| --- | --- | --- | --- |
| Male (%) | 243 (57.4) | 99 (63.9) | 0.164 |
| Age (years) | 37.0 (30.0-44.0) | 37.0 (30.0-46.0) | 0.616 |
| BMI (kg/m^2^) | 24.5 (22.0-27.1) | 25.0 (22.5-27.9) | 0.112 |
| MAP (mmHg) | 97.3 (89.3-106.7) | 99.0 (90.0 -106.7) | 0.771 |
| Total serum protein (g/L) | 65.5 (60.6-70.3) | 64.3 (59.5-69.4) | 0.294 |
| ALB (g/L) | 39.3 (36.0 -42.1) | 39.0 (36.5 -42.1) | 0.860 |
| Blood urea nitrogen (mmol/L) | 5.7 (4.5-7.0 ) | 6.0 (4.5-7.8) | 0.108 |
| SUA (umol/L) | 372.5 (305.6-449.8) | 410.1 (313.5-456.4) | 0.133 |
| Total cholesterol (mmol/L) | 4.3 (3.7-5.0) | 4.3 (3.8-5.0) | 0.936 |
| Triglycerides (mmol/L) | 1.6 (1.1-2.2) | 1.7 (1.3-2.5) | 0.059 |
| HDL-C (mmol/L) | 1.1 (0.9-1.3) | 1.0 (0.8-1.3) | 0.425 |
| LDL-C (mmol/L) | 2.7 (2.3-3.3) | 2.8 (2.2-3.2) | 0.738 |
| Hemoglobin (g/L) | 134.0 (119.0-148.0) | 133.0 (123.0-145.0) | 0.814 |
| URBC (HPF) | 9.0 (3.5-22.5) | 6.5 (3.0-17.5) | 0.096 |
| Serum creatinine (umol/L) | 99.5 (77.0 -126.0) | 101.1 (77.1-147.1) | 0.297 |
| eGFR (ml/min per 1.73 m^2^) | 80.6 (57.2 -103.0) | 76.1 (50.7-103.3) | 0.275 |
| CKD (%) |  |  | 0.090 |
| 1 | 160 (37.8) | 53 (34.2) |  |
| 2 | 145 (34.3) | 55 (35.5) |  |
| 3a | 64 (15.1) | 15 (9.7) |  |
| 3b | 37 (8.8) | 20 (12.9) |  |
| 4 | 17 (4.0) | 12 (7.7) |  |
| 24h urinary protein (g/24h) | 1.2 (0.7-2.0) | 1.4 (0.7-2.4) | 0.117 |
| Oxford classification (%) |  |  |  |
| M1 | 185 (43.7) | 73 (47.1) | 0.471 |
| E1 | 84 (19.9) | 33 (21.3) | 0.704 |
| S1 | 310 (73.3) | 102 (65.8) | 0.078 |
| T |  |  | 0.508 |
| T1 | 116 (27.4) | 50 (32.3) |  |
| T2 | 81 (19.1) | 29 (18.7) |  |
| C |  |  | 0.148 |
| C1 | 114 (27.0) | 37 (23.9) |  |
| C2 | 5 (1.2) | 0 (0) |  |

IQR: interquartile range; BMI, body mass index; MAP: mean arterial pressure; ALB: albumin; SUA: serum uric acid; HDL-C, High-density lipoprotein cholesterol; LDL-C, low-density lipoprotein cholesterol; Hb: hemoglobin; URBC: urine red blood cell count; eGFR, estimated glomerular filtration rate; CKD, chronic kidney disease; M, mesangial hypercellularity; E, endocapillary hypercellularity; S, segmental glomerulosclerosis or adhesion; T, tubular atrophy/interstitial fibrosis; C, cellular/fibrocellular crescents.

TABLE S2: Distribution of syndrome elements in the 423 patients included and 155 patients followed＜90 days (n (%)).

| syndrome elements | n=423 | n=155 | *P* |
| --- | --- | --- | --- |
| Heart | 30 (7.1) | 11 (7.1) | 0.998 |
| Liver | 212 (50.1) | 80 (51.6) | 0.750 |
| Spleen | 154 (36.4) | 67 (43.2) | 0.135 |
| Lung | 46 (10.9) | 21 (13.5) | 0.374 |
| Kidney | 371 (87.7) | 139 (89.7) | 0.515 |
| Stomach | 10 (2.4) | 5 (3.2) | 0.778 |
| Jifu | 7 (1.7) | 2 (1.3) | 1.000 |
| Biao | 8 (1.9) | 3 (1.9) | 1.000 |
| Qi-deficiency | 204 (48.2) | 80 (51.6) | 0.471 |
| Blood-deficiency | 136 (32.2) | 60 (38.7) | 0.140 |
| Yin-deficiency | 334 (79.0) | 113 (72.9) | 0.123 |
| Yang-deficiency | 147 (34.8) | 63 (40.6) | 0.192 |
| Qi-depression | 36 (8.5) | 12 (7.7) | 0.767 |
| Blood-stasis | 65 (15.4) | 25 (16.1) | 0.823 |
| Dampness | 154 (36.4) | 60 (38.7) | 0.611 |
| Heat | 53 (12.5) | 16 (10.3) | 0.468 |
| Phlegm | 144 (34.0) | 65 (41.9) | 0.080 |
| Water retention | 37 (8.7) | 21 (13.5) | 0.089 |
| Yang hyperactivity | 23 (5.4) | 7 (4.5) | 0.658 |

TABLE S3: Distribution of CKD stages and 24h urinary protein for patients at the time of renal biopsy with Yang-deficiency and non-Yang deficiency [median (IQR)/ n (%)]

| Characteristics | Non-Yang-deficiency (n=276) | Yang-deficiency (n=147) | *P* |
| --- | --- | --- | --- |
| eGFR (ml/min per 1.73 m^2^) | 80.7(59.0-104.0) | 79.0(54.8-101.6) | 0.479 |
| CKD, n (%) |  |  | 0.811 |
| 1 | 105 (38.0) | 55 (37.4) |  |
| 2 | 99 (36.0) | 46 (31.3) |  |
| 3a | 39 (14.1) | 25 (17.0) |  |
| 3b | 23 (8.3) | 14 (9.5) |  |
| 4 | 10 (3.6) | 7 (4.8) |  |
| 24h urinary protein (g/24h) | 1.2 (0.7, 1.9) | 1.3 (0.8, 2.4) | 0.106 |
| 24h urinary protein grading, n (%) |  |  | 0.379 |
| ＜1g/24h | 118(42.7) | 53(36.0) |  |
| 1-3.5 g/24h | 134(48.6) | 78(53.1) |  |
| ≥3.5 g/24h | 24(8.7) | 16(10.9) |  |

eGFR, estimated glomerular filtration rate; CKD, chronic kidney disease.
